# Visfatin is involved in promotion of colorectal carcinoma malignancy through an inducing EMT mechanism

**DOI:** 10.18632/oncotarget.8615

**Published:** 2016-04-06

**Authors:** Jing Yang, Kun Zhang, Haixing Song, Mingbo Wu, Jingyi Li, Ziyi Yong, Sheng Jiang, Xi Kuang, Tao Zhang

**Affiliations:** ^1^ School of Biomedical Sciences, Chengdu Medical College, Chengdu, China; ^2^ School of Pharmacy, Chengdu Medical College, Chengdu, China; ^3^ School of Basic Medical Sciences, Chengdu Medical College, Chengdu, China; ^4^ Department of Pharmacology, Key Laboratory of Drug Targeting and Drug Delivery Systems, West China School of Pharmacy, Sichuan University, Sichuan, China

**Keywords:** visfatin, colorectal cancer, EMT, Snail, Akt/GSK-3β

## Abstract

Increasing evidences suggested visfatin, a newly discovered obesity-induced adipocytokine, is involved in promotion of cancer malignancy and correlated with worse clinical prognosis. While its effects and mechanisms on progression of colorectal cancer (CRC) remain unclear. Our clinical data show that visfatin protein is over expressed, positive associated with lymph node metastasis, high-grade tumor, and poor prognosis in 87 CRC patients. The levels of plasma visfatin are significantly upregulated in Stage IV colon cancer. Visfatin can significantly promote the *in vitro* migration and invasion of CRC cells via induction epithelial mesenchymal transition (EMT). It can increase the expression and nuclear translocation of Snail, a key transcription factor in regulating EMT. While silencing of Snail attenuates visfatin induced EMT. Further studies reveal visfatin can inhibit the association of Snail with GSK-3β and subsequently suppress ubiquitylation of Snail. In addition, visfatin can increase the expression and nuclear translocation of β-catenin, elevate its binding with Snail promoter, and then increase the transcription of Snail. While inhibitor of PI3K/Akt, LY294002, abolishes visfatin induced up regulation of Snail, Vimentin (Vim), β-catenin, and phosphorylated GSK-3β. In summary, our data suggest that increased expression of visfatin are associated with a more aggressive phenotype of CRC patients. It can trigger the EMT of CRC cells via Akt/GSK-3β/β-catenin signals.

## INTRODUCTION

The investigation of underlying pathological mechanisms of cancer invasion and metastasis is important to improve the cure rate and life quality of colorectal cancer (CRC) patients. The adipokine visfatin, which is also indentified as the pre-B-cell colony-enhancing factor (PBEF) and nicotinamide phosphoribosyltransferase (NAMPT), is predominantly secreted by visceral fat tissue, particularly by the macrophages [1, 2]. Studies indicated that visfatin can also enter into the cytosol and nucleus and then promote the progression of many biological behaviors such as inflammation, angiogenesis, energy metabolism, and cell longevity [3–6]. Recently, visfatin has been reported to be associated with tumorigenesis and/or metastasis of many human cancers such as colon, stomach, brain, pancreas, liver, prostate, and breast cancers [7]. Higher visfatin levels in breast cancer are correlated with overall survival and disease-free survival [8]. Literatures also reported that visfatin overexpression can predict poor response of the patients to doxorubicin [9] or fluorouracil [10] based chemotherapy. Visfatin is closely associated with the pathogenesis of colon cancer, therefore it is considered as a novel and promising biomarker of CRC [11]. The visfatin levels in patients with advanced and early CRC cancer were higher than in controls (least significant difference test, P=0.004 and 0.013, respectively) [12]. Therefore, understanding the effects and mechanisms of visfatin on progression of CRC will be important for the development of prevention and treatment strategies.

Epithelial–mesenchymal transition (EMT), which refers to the epithelial cells get the characteristics of mesenchymal cells under specific physiological conditions, is considered as the first and key step for the migration and invasion of cancers including CRC [13]. Cells undergone of EMT will loss cell-cell adhesion and gain migratory and invasive traits. The loss or decrease of E-Cadherin (E-Cad) is considered to be the primary and most important marker of EMT [14]. Transcription factors such as Snail, Slug, Twist and Zeb, which can suppress the expression of E-Cad directly or indirectly, can regulate the progression of EMT [15]. The process of EMT is positively correlated with the progression, metastasis, and drug resistance of CRC [16]. Increasing evidences suggested that visfatin can promote the migration and invasion of breast [17] and ovarian [18] cancer cells. One recent study revealed that visfatin can enhance the migration and invasion of osteosarcoma cells [19]. However, the effects and mechanisms of visfatin on progression and EMT of CRC cells as a cytokine remain unclear.

Our present study revealed that the expression of visfatin is significantly negatively correlated with the prognosis of CRC patients. Further, *in vitro* studies suggested that visfatin can promote the EMT phenotype of CRC cells via up regulation of Snail through Akt/GSK-3β/β-catenin signals. Our findings indicated that visfatin might be a target to develop anti-cancer drugs for CRC patients.

## RESULTS

### The expression profile of visfatin and its correlation with clinicopathologic characteristics of CRC patients

**Figure 1:**
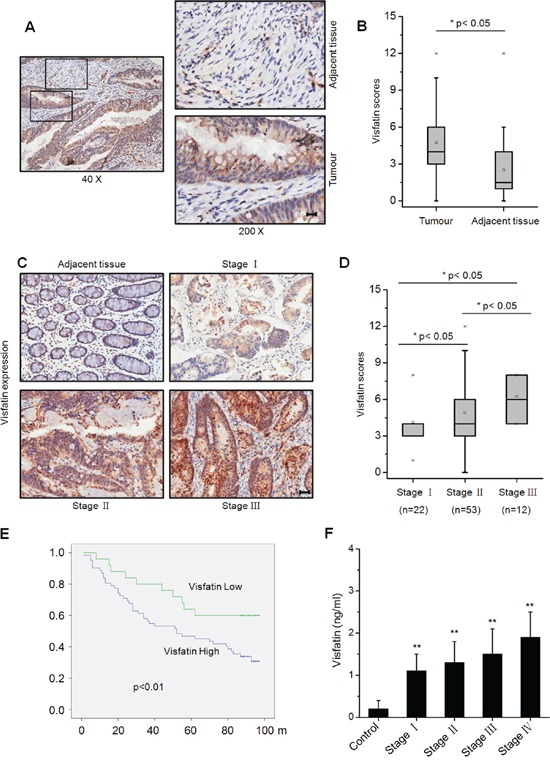
Visfatin expression in clinical samples of colorectal cancer patients **A.** Typical immunohistochemical staining for visfatin expression in a tumor and its adjacent tissue from a CRC patient, scale bar is 20 μm; **B.** Scores for visfatin staining in paired, adjacent normal and tumor tissue samples obtained from different CRC patients by immunohistochemical staining; **C.** Typical staining for visfatin in tumor samples from CRC patients with different TNM stages, using immunohistochemical staining, scale bar is 100 μm; **D.** Scores for visfatin staining in tumor samples from CRC patients with different TNM stages by immunohistochemical staining; **E.** Visfatin expression is negatively correlated with prognosis in CTC patients. Overall survival (OS) in patients with high/medium levels of visfatin (n=62) vs the remaining patients (n=25) was plotted by the Kaplan-Meier method; **F.** The content of visfatin (ng/ml) in health (n=200) and different TNM stages CRC patients (n=50 for each stage).

To date there are very limited data about visfatin and clinical prognosis of CRC patients. Therefore we assessed the clinical roles of visfatin in CRC by analyzing the protein levels of visfatin in one commercial tissue microarray from 87 CRC patients. We found that the expression of visfatin was stronger in most primary CRC tissue samples than in their normal counterparts (Figure 1A & 1B), and elevated visfatin expression can be detected in CRC patients at advanced clinical stage [assessed using the tumor node metastasis (TNM) system] (Figure 1C & 1D). Kaplan-Meier analysis of all 87 patients demonstrated a statistically significant negative correlation between overall survival (OS) and visfatin expression level (*p*<0.001) (Figure 1E). Further, statistical analysis revealed that patients whose tumors express increased node metastasis had greater visfatin expression compared with those with low levels of node metastasis (p<0.05, Table 1). We next analyzed the plasma visfatin levels in cancer patients and normal controls. Our results revealed that the concentration of visfatin in health people was significantly (p<0.01) less than that in CRC patients (Figure 1F). Spearman correlation analysis showed that the plasma visfatin levels were highly correlated with colon cancer stages (r =0.505, p<0.05). After stratification of the cancer patients according to their clinical stages, plasma visfatin was significantly upregulated in Stage IV colon cancer when compared with that in normal controls, Stage I–II, Stage III, and Stage I–III combined. Collectively, our data suggested that increased expression of visfatin resulted in a more aggressive phenotype in CRC patients.

**Table 1:** Visfantin expression in 87 CRC patients

Characteristics	N	Visfatin Low/No	Visfatin High/Medium	*p* value
**Age**				
≤60	16	6	10	0.391
>60	71	19	52	
**Gender**				
Male	46	12	34	0.563
Female	41	13	28	
**Tumor size**				
≤10 cm^3^	29	8	21	0.866
>10 cm^3^	58	17	41	
**Node metastasis (n=78)**				
Negative(<10)	24	12	12	0.023
Positive (≥10)	54	13	41	

### Visfatin triggers the *in vitro* motility and EMT of CRC cells

**Figure 2:**
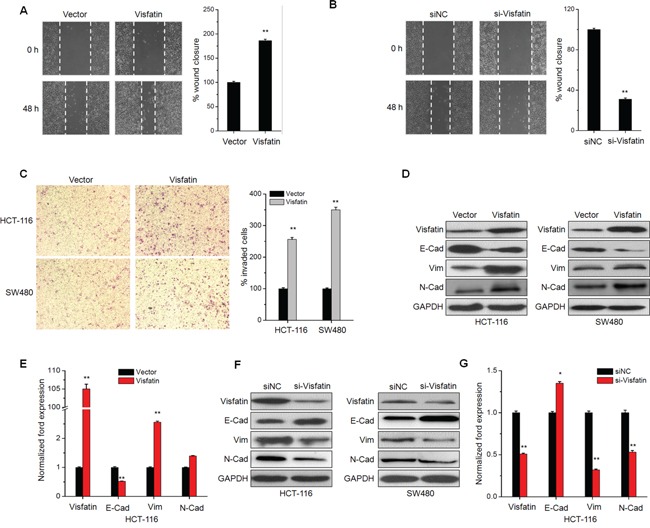
Visfatin triggers the *in vitro* motility and EMT of CRC cells **A.** Confluent monolayers of HCT-116 cells transfected with vector control or visfatin construct for 24 h were scraped by a pipette tip to generate wounds and then were cultured for 24 h. Representative images of wounds and the statistic results at each concentration were recorded. **B.** Would healing assays for HCT-116 cells transfected with siNC or si-visfatin for 24 h; **C.** HCT-116 or SW480 cells were transfected with vector control or visfatin construct for 24 h and then allowed to invade spread through the matrix gel and into the under-side of the filter for 24 h. The number of invaded cells were fixed, stained, photographed, and compared with the control group. **D** and **F.** HCT-116 or SW480 cells were transfected with vector control, visfatin construct, siNC control or si-visfatin for 48 h as indicated, then the protein levels of visfatin, E-cad, Vim, and N-Cad were analyzed by Western blot analysis. **E** and **G.** HCT-116 cells were transfected with visfatin construct or si-visfatin for 24 h as indicated the mRNA levels of visfatin, E-cad, Vim, and N-Cad were analyzed by qRT-PCR. Scale bar is 100 μm, * p<0.05 compared with control, ** p<0.01 compared with control.

Clinical data revealed that visfatin is positively correlated with lymph node metastasis of CRC patients, we then investigated the effects of visfatin on the *in vitro* motility of CRC cells. Overexpression of visfatin significantly promoted wound closure of HCT-116 cells (Figure 2A). While the silencing of visfatin by its specific siRNA inhibited the wound closure as compared to the control group (Figure 2B). Boyden chambers were used to confirm the roles of visfatin on the *in vitro* invasion of CRC cells. As shown in Figure 2C, the number of invaded HCT-116 and SW480 cells transfected with visfatin was significantly (p<0.01) greater than that of the control cells.

The increased motility of CRC cells suggested visfatin may modulate the EMT process, of which the epithelial makers E-cad is down-regulated, whereas the mesenchymal markers N-cad and Vim are up-regulated [20]. The EMT of CRC cells was observed after stimulation with visfatin for 48 h. Cells resulted in a significant change in morphology, from cobblestone morphology to mesenchymal spindle-like and fusiform features (Figure S1). Over expression of visfatin obviously down regulated the expression of E-Cad, while increased the expression of Vim and N-Cad in both HCT-116 and SW480 cells (Figure 2D). Similarly, results of mRNA expression profiles of EMT markers in HCT-116 cells also confirmed that over expression of visfatin triggered EMT (Figure 2E). Further, the silencing of visfatin by siRNA exhibited opposite effects in HCT-116 cells (Figure 2F & 2G). Collectively, these observations showed a critical role of visfatin in the EMT and metastatic phenotypes of CRC cells.

### Visfatin triggers the EMT of CRC cells via up regulation of Snail

**Figure 3:**
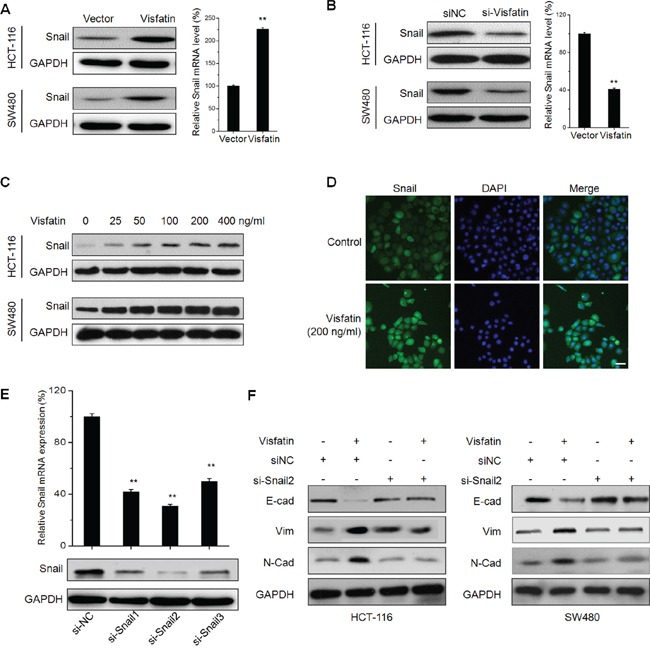
Visfatin triggers the EMT of CRC cells via up regulation of Snail **A.** HCT-116 or SW480 cells were transfected with vector control or visfatin construct for 24 h, then the protein or mRNA levels of Snail were analyzed by Western blot analysis or RT-PCR, respectively; **B.** HCT-116 or SW480 cells were transfected with siNC or si-visfatin for 24 h, then the protein or mRNA levels of Snail were analyzed by Western blot analysis or RT-PCR, respectively; **C.** HCT-116 or SW480 cells were treated with increasing concentrations of visfatin for 24 h, the levels of Snail were checked by Western blot analysis; **D.** HCT-116 cells were treated with 200 ng/ml visfatin for 24 h, the cellular location of Snail (green) were examined by immunofluorescence staining and nuclei were stained with DAPI (blue), scale bar is 20 μm; **E.** HCT-116 cells were transfected with Snail specific si-RNA (si-Snail1~3) or negative control si-RNA (si-NC) for 24 h, and then the mRNA and protein expression of Snail were analyzed by qRT-PCR and Western-blot, respectively; **F.** HCT-116 or SW480 cells transfected with Snail specific si-RNA (si-Snail2) or negative control si-RNA (si-NC) for 24 h and then exposed to 200 ng/ml visfatin for another 48 h, the expression of EMT related markers were measured by use of Western blot analysis. **p < 0.01 compared with control.

Since transcription factors Snail plays a critical role in regulating EMT [21], we then investigated whether visfatin can promote the expression and nuclear translocation of Snail. Results showed that both protein and mRNA expression of Snail were significantly up regulated in CRC cells transfected with visfatin construct (Figure 3A). While, the protein and mRNA expression of Snail were obviously down regulated in HCT-116 and SW480 cells transfected with si-visfatin (Figure 3B). Western blot analysis showed that visfatin can increase the expression of Snail in CRC cells via a dose dependent manner (Figure 3C). Further, the sub cellular localization of Snail in visfatin (200 ng/ml) treated HCT-116 cells were checked by use of immunofluorescence staining. The result showed that visfatin treatment obviously triggered the nuclear translocation of Snail in HCT-116 cells (Figure 3D).

To verify the roles of Snail in visfatin induced EMT of CRC cells, HCT-116 and SW480 cells were transfected with non-targeting control si-RNA or si-Snail for 24 h, and then treated with visfatin (200 ng/ml) for 48 h. The silencing of Snail was confirmed by qRT-PCR and Western blot analysis (Figure 3E). The results revealed that silencing of Snail by both si-Snail2 (Figure 3F) and si-Snail1 (Figure S2) significantly attenuated visfatin induced down regulation of E-cad and up regulation of Vim and N-Cad in CRC cells. It suggested that Snail is essential for visfatin induced EMT in CRC cells.

### Akt/ GSK-3β/β-catenin signals are crucial for visfatin induced Snail up regulation

**Figure 4:**
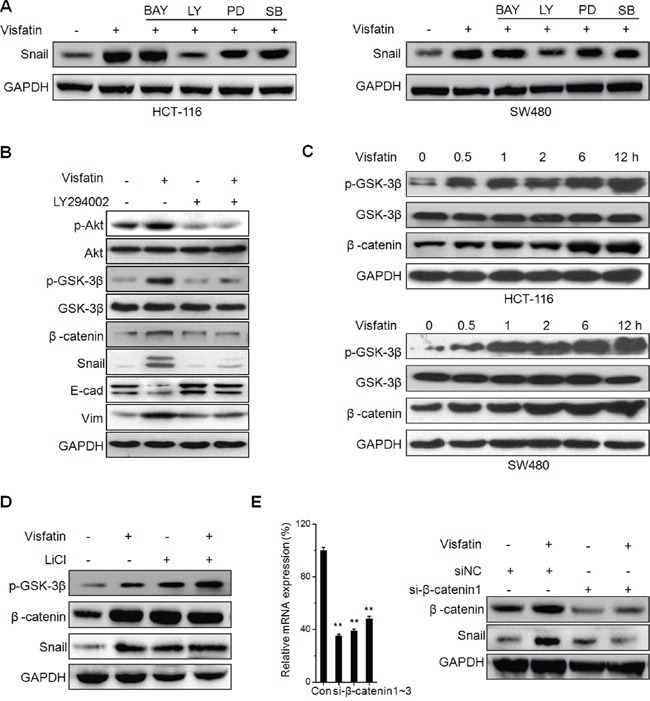
Akt/ GSK-3β/β-catenin signals are crucial for visfatin induced Snail up regulation **A.** HCT-116 or SW480 cells were pretreated with inhibitors of NF-κB (BAY11-7082, BAY), PI3K/AKT (LY294002, LY), ERK1/2 (PD98059, PD), and p38 (SB-203580, SB) for 1 h, and then treated with visfatin for 12 h; **B.** HCT-116 cells were pretreated with LY294002 for 1 h, and then treated with visfatin for 12 h; **C.** HCT-116 or SW480 cells were treated with visfatin for increasing time periods, the expression of p-GSK-3β and β-catenin were checked by Western blot analysis; **D.** HCT-116 cells were pretreated with LiCl for 1 h, and then treated with visfatin for 12 h; **E.** HCT-116 cells were transfected with si-β-catenin1 or si-NC for 24 h, and then treated with visfatin for 12 h. **p < 0.01 compared with control.

To investigate the molecular mechanisms underlying visfatin induced Snail up regulation, inhibitors of NF-κB (BAY11-7082), PI3K/AKT (LY294002), ERK1/2 (PD98059), and p38 (SB-203580) were used, since visfatin has been reported to activate these signals [22]. The results showed that PI3K/AKT inhibitor (LY294002), while not the others, obviously blocked visfatin induced Snail expression (Figure 4A). It suggested that activation of the PI3K/AKT pathway is responsible for visfatin mediated Snail over expression.

GSK-3β/β-catenin signals which act as the downstream of PI3K/AKT pathway can regulate Snail during EMT [23–25]. GSK-3β maintains an active state in dephosphorylated form, while β-catenin is complexed with and phosphorylated by GSK-3β for the ubiquitination and the following proteasome mediated degradation [25]. To investigate whether GSK-3β/β-catenin signals were involved in visfatin induced Snail up regulation, we treated HCT-116 cells with LY294002 prior to visfatin treatment, and then the expression of p-AKT(Ser473), p-GSK-3β (Ser9), β-catenin, and Snail were determined by Western blot analysis. The results showed that the levels of p-AKT, p-GSK-3β, β-catenin, and Snail were increased after visfatin treatment at 6 h, while these effects were reversed upon treating with LY294002 alone or in combination with visfatin (Figure 4B). Further, we found that visfatin treatment can increase the levels of p-GSK-3β and β-catenin in both HCT-116 and SW480 cells via a time dependent manner (Figure 4C). To further verify the roles of GSK-3β, we pretreated HCT-116 cells with or without LiCl (a potent GSK-3b inhibitor) for 1 h and then further treated with visfatin for 6 h, the results showed that inhibition of GSK-3β markedly elevated the levels of Snail expression, while visfatin induced Snail up regulation was not further elevated in the presence of LiCl (Figure 4D). Further, we knocked down the expression of β-catenin in HCT116 cells. The results showed that both si-β-catenin1 (Figure 4E) and si-β-catenin2 (Figure S3) significantly attenuated visfatin induced Snail up regulation. This was confirmed by the results of immunofluorescence staining that visfatin treatment can increase the expression and nuclear localization of β-catenin in HCT-116 Cells (Figure S4). Taken together, these results demonstrated that visfatin up-regulated Snail in CRC cells by activating Akt/GSK-3β/β-catenin signaling.

### The roles of GSK-3β/β-catenin in visfatin induced Snail up regulation

**Figure 5:**
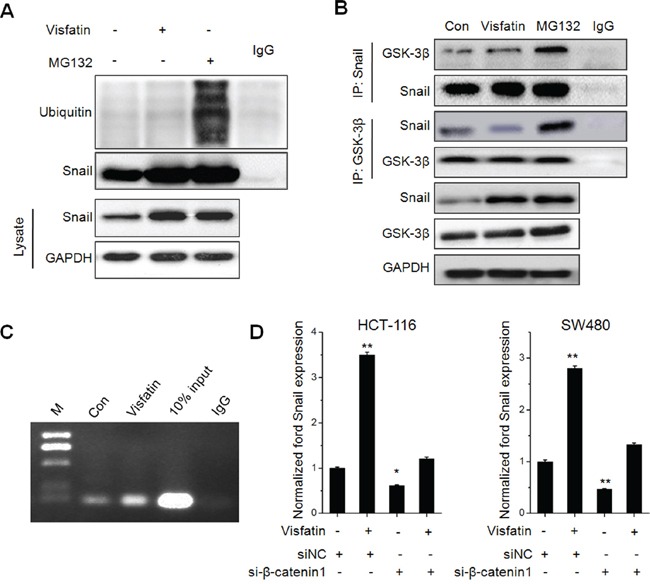
The roles of GSK-3β/β-catenin in visfatin induced Snail up regulation **A.** HCT116 cells were treated with visfatin (200 ng/ml) or MG132 (10 mM) for 12 h, After Snail was immunoprecipitated from equal amount of lysates (two lower panels), the ubiquitination of Snail was examined by Western blot analysis; **B.** HCT116 cells were treated with visfatin (200 ng/ml) or MG132 (10 mM) for 12 h, Snail or GSK-3β were immunoprecipitated respectively from equal amount of lysates and the associated GSK-3β or Snail were detected by Western blot analysis; **C.** HCT-116 cells were treated with visfatin (200 ng/ml) or MG132 (10 mM) for 12 h, and then the recruitment of β-catenin to Snail promoter was determined by ChIP. Immunoprecipitated products were amplified by Real-time PCR; **D.** HCT-116 or SW480 cells were transfected with siNC or si-β-catenin1 for 24 h, and then further treated with visfatin (200 ng/ml) for 12 h, the mRNA levels of Snail were measured by Real-time PCR. *p < 0.05 compared with control; **p < 0.01 compared with control.

Recent studies indicated GSK-3β/β-catenin can up regulate Snail expression via transcriptional and post-transcriptional modification [26, 27]. Considering that ubiquitin mediated proteasomal degradation processes are critical for protein stability of Snail, we measured the ubiquitination state of Snail in HCT-116 cells treated with visfatin or proteasome inhibitor MG132 for 12 h. The results showed that visfatin obviously suppressed the ubiquitylation of Snail as compared with MG132, although total stabilized Snail proteins were parallel (Figure 5A). Previous studies indicated that GSK-3β can phosphorylate Snail and then induce the protein degradation of Snail [23], then we examined the association of Snail and GSK-3β. The results showed that the association of Snail with GSK-3β was decreased in cells treated with visfatin, compared with cells treated with MG-132 (Figure 5B). Similarly, when GSK-3β was immunoprecipitated from HCT116 cells, the associated Snail was markedly decreased in cells treated with visfatin as compared with MG-132. These results suggested that visfatin can inhibit the association of Snail with GSK-3β and subsequently suppressed ubiquitylation of Snail.

It was suggested that β-catenin can directly activate the transcription of Snail [27]. Then, we determined if visfatin treatment influenced the binding of β-catenin subunits on the *SNAIL* promoter. ChIP assays showed that significant β-catenin occupancy at Snail promoter when treated with visfatin (Figure 5C). Further, the silencing of β-catenin decreased the transcription of Snail and attenuated visfatin induced up regulation of Snail transcription in HCT-116 cells (Figure 5D). Collectively, our data revealed that visfatin can increase the expression of β-catenin, elevate its binding with Snail promoter, and then increase the transcription of Snail.

## DISCUSSION

Increased evidences suggested visfatin can promote the development of CRC and other cancers such as breast, prostate, and endometrial cancers [28, 29]. Recently, limited literatures indicated that visfatin might promote the EMT of cancer cells as a soluble factor independent of its enzymatic activity [19, 30]. Our present study revealed that increased expression of visfatin resulted in a more aggressive phenotype in CRC patients. Further, visfatin can increase the *in vitro* migration and invasion of CRC cells via induction of EMT. Snail, an important transcription factor of EMT, mediated visfatin induced EMT of CRC cells. Akt/ GSK-3β/β-catenin signals were crucial for visfatin induced Snail up regulation. Generally, the present study revealed that visfatin can be considered as a therapy target for CRC.

To the best of our knowledge, this is the first systematic study to evaluate the prognostic significance of visfatin expression in CRC patients. A significant increase in visfatin expression was observed in colorectal cancer tissues. Furthermore, visfatin is an indicator of poor prognosis in colorectal cancer for both plasma concentrations and immunohistochemical staining of tumor biopsies. As a newly discovered obesity-induced adipocytokine, Dalamaga et al. observed that the mean serum visfatin level was significantly higher in breast cancer than in controls or patients with benign breast lesions [31]. As to CRC, the visfatin levels in patients with advanced and early CRC cancer were higher than in controls [12]. The plasma visfatin levels can provide potential biomarkers for predicting early and advanced CRC and yield a ROC curve area of 72 and 86%, respectively [12]. This was supported by other studies that visfatin levels were significantly higher in CRC patients than that in controls (p<0.01) [11, 32]. Our present study revealed that primary tumor tissue visfatin level was associated with the clinicopathologic stage. Generally, our observation is in agreement with previous reports that higher visfatin expression is observed in primary colorectal cancer than in nonneoplastic mucosa.

Our study revealed that visfatin treatment can trigger the EMT of CRC cells via up regulation of Snail. Soncini et al showed visfatin over expression is sufficient to induce EMT in mammary epithelial cells via elevation the expression of TGF-β [30]. Visfatin also can promote the EMT of osteosarcoma cells via the NF-κB/Snail-1/EMT pathway [19]. Several transcription factors have been implicated in the progression of EMT. Our present study revealed that visfatin treatment obviously increased the expression and nuclear translocation of Snail in CRC cells, while Snail knockdown attenuated visfatin induced EMT. It confirmed that Snail is essential for visfatin induced EMT in CRC cells. This is also consistent with recent study that visfatin can increase both protein an mRNA levels of Snail in osteosarcoma cells [19].

Several downstream pathways including PI3K/Akt and NF-κB have been reported to contribute to visfatin-induced targeted gene transcription [28, 33]. We found that the inhibitor of PI3K/Akt, while not NF-κB, ERK1/2, or p38-MAPK, attenuated visfatin induced up regulation of Snail and EMT progression. The activation of Akt/GSK-3β signal has been reported to be associated with a loss of cell adhesion, increased of cell motility, and poor prognosis of CRC [34]. GSK-3β can decrease the ubiquitination and proteasomal degradation of Snail [23, 35]. Further, inactivation of GSK-3β can upregulate β-catenin during gastrin [36] and TNF-α [37] induced EMT of cancer cells. Our study revealed that visfatin can up regulate the expression of Snail via both transcriptional and post-transcriptional modification though Akt/GSK-3β/β-catenin signals. Firstly, visfatin increases Snail expression by increasing Snail protein stability particularly through phosphorylation of GSK-3β and then inhibition their association. Secondly, visfatin can elevate the binding of β-catenin subunits on the SNAIL promoter and then promote the transcription of Snail [27]. Although the exact role of GSK-3β/β-catenin in cancer metastasis remains controversial and is likely cell type- and stimulus-dependent, our study revealed that Akt/GSK-3β/β-catenin is crucial for visfatin induced EMT of CRC cells.

In summary, we have identified that visfatin can trigger the EMT of CRC cells via Akt/GSK-3β/β-catenin signals and suggested that increased expression of visfatin resulted in a more aggressive phenotype in CRC patients. Though the precise mechanism remains undetermined, these findings will help to better understand the roles of visfatin on the progression of CRC and indicate that visfatin might be a valuable target for CRC therapy.

## MATERIALS AND METHODS

### Patients plasma samples and visfatin measurement

The study was approved by the Ethical Committee of Chengdu Medical College. Plasma samples were collected to investigate the concentration of plasma visfatin with the clinicopathologic characteristics of CRC patients. 200 plasma samples from CRC patients and 100 plasma samples from healthy individuals were obtained from The First Affiliated Hospital of Chengdu Medical College (Sichuan province, China) between 2005 to 2012. Pathologic classification of disease in all patients was performed following the International Union Against Cancer (UICC) and American Joint Committee on Cancer (AJCC) TNM staging system for colon cancer established in 2003. Blood samples were collected from all patients before operation and therapy. Visfatin levels in plasma were measured by standard enzyme immunoassay.

### Immunohistochemistry and scoring

Commercial tissue microarray (TMAs; HCol-Ade180Sur-06, Shanghai Outdo Biotech, Shanghai, China) from 90 CRC patients with paired normal mucosal counterparts were used to evaluate the expression of visfatin protein. Immunohistochemistry using a visfatin antibody was performed as previously described [38]. Briefly, antigen retrieval was performed using high pressure for 5 min, using citrate buffer, pH 6.0. The sections were incubated overnight with the primary antibodies at 4°C. Negative controls were performed by replacing the primary antibody with PBS. Finally, the slides were analyzed separately by two pathologists without knowing the patients' clinical information. The staining intensity was scored on a scale of 0–3 as negative (0), weak (1), medium (2) or strong (3). The extent of the staining, defined as the percentage of positive staining areas of tumor cells in relation to the whole tumor area, was scored on a scale of 0 (0%), 1 (1–25%), 2 (26–50%), 3 (51–75%) and 4 (76–100%). An overall protein expression score (overall score range, 0–12) was calculated by multiplying the intensity and positivity scores according to previous study [39].

### Cell culture and transfection

Human CRC cell HCT-116 and SW480 cells were purchased from the American Type Culture Collection (Manassas, VA, USA), maintained in our laboratory, and cultured in RPMI 1640 or DMEM medium (Invitrogen Corporation, Carlsbad, CA, USA) supplemented with 10% heat-inactivated fetal Bovin serum, 100 U/ml penicillin, and 10 μg/ml streptomycin at 37°C in a 5% CO_2_ atmosphere. An ABI 3130 Genetic Analyzer (Applied Biosystems) was used for the profiling. The DNA profile data was cross-checked with the ATCC data bank. For transfection, cells were seeded into plates and transfected with pEGFP-N1 (vector control), pEGFR-N1/visfatin (Visfatin construct), siRNA negative control (si-NC), si-Snail, si-visfatin, or si-β-catenin by use of Lipofectamine 2000 reagent (Invitrogen).

### *In vitro* wound-healing and transwell invasion assay

A wound-healing assay was used to compare the migratory ability of HCT-116 and SW480 cells as described previously [40]. The cell migration and invasion assay was using 6-well transwell plates (Falcon cell culture inserts, 8-μm pore size, BD, NJ) according to previous study [41].

### Western blotting analysis

Western blotting was performed as previously described [42].

### Quantitative real-time PCR

After treatment as indicated, total mRNA of cells was extracted with TRIZOL reagent. First strand of cDNA was generated from 2 μg total RNA using oligo-dT primer and Superscript II Reverse Transcriptase (GIBCO BRL, Grand Island, NY, USA). Quantitative Real-Time PCR was run on an iCycler (Bio-Rad, Hercules, USA) using validated primers and SYBR Premix Ex Taq II (Takara, Japan) for detection. The cycle number when the fluorescence first reached a preset threshold (Ct) was used to quantify the initial concentration of individual templates for expression of mRNA of genes of interest. Transcripts of the housekeeping gene GAPDH in the same incubations were used for internal normalization. Primer pairs were as follows: visfatin, forward 5b′- GCC AGC AGG GAA TTT TGT TA-3′ and reverse 5′- TGA TGT GCT GCT TCC AGT TC-3′; E-Cad forward 5′- TAC ACT GCC CAG GAG CCA GA -3′ and reverse 5′- TGG CAC CAG TGT CCG GAT TA -3′; Vim forward 5′- TGA GTA CCG GAG ACA GGT GCA G -3′ and reverse 5′- TAG CAG CTT CAA CGG CAA AGT TC-3′; N-Cad forward 5′- GAC GGT TCG CCA TCC AGA C-3′ and reverse 5′- TCG ATT GGT TTG ACC ACG G-3′; Snail, forward 5′- GAC CAC TAT GCC GCG CTC TT -3′ and reverse 5′- TCG CTG TAG TTA GGC TTC CGA TT -3′; β-Catenin, forward 5′- TCC CTG AGA CGC TAG ATG AGG -3′ and reverse 5′- CGT TTA GCA GTT TTG TCA GCT C -3′; GAPDH, forward 5′-GCA CCG TCA AGG CTG AGA AC-3′ and reverse 5′-TGG TGA AGA CGC CAG TGG A-3′.

### Immunofluorescence

Immunofluorescence staining was carried out as described previously [43].

### Statistical analysis

All values were reported as mean ± SD of three independent experiments unless otherwise specified. Data were analyzed by two-tailed unpaired Student's t-test between two groups and by One-Way ANOVA followed by Bonferroni test for multiple comparison involved. The survival curves were plotted by using Kaplan–Meier analysis. Statistical analysis was carried out using SPSS 16.0 for Windows. A *p*-value of < 0.05 was considered to be statistically significant.

## SUPPLEMENTARY FIGURES


