# Supplementary material for: Visfatin is involved in promotion of colorectal carcinoma malignancy through an inducing EMT mechanism
Source: Oncotarget. 2016 Apr 6;7(22):32306–17. doi: 10.18632/oncotarget.8615 (PMC5078014; doi:10.18632/oncotarget.8615)
Supplement: Supplementary file 1 [file oncotarget-07-32306-s001.pdf]

Visfatin is involved in promotion of colorectal carcinoma malignancy through an inducing EMT mechanism

SUPPLEMENTARY FIGURES

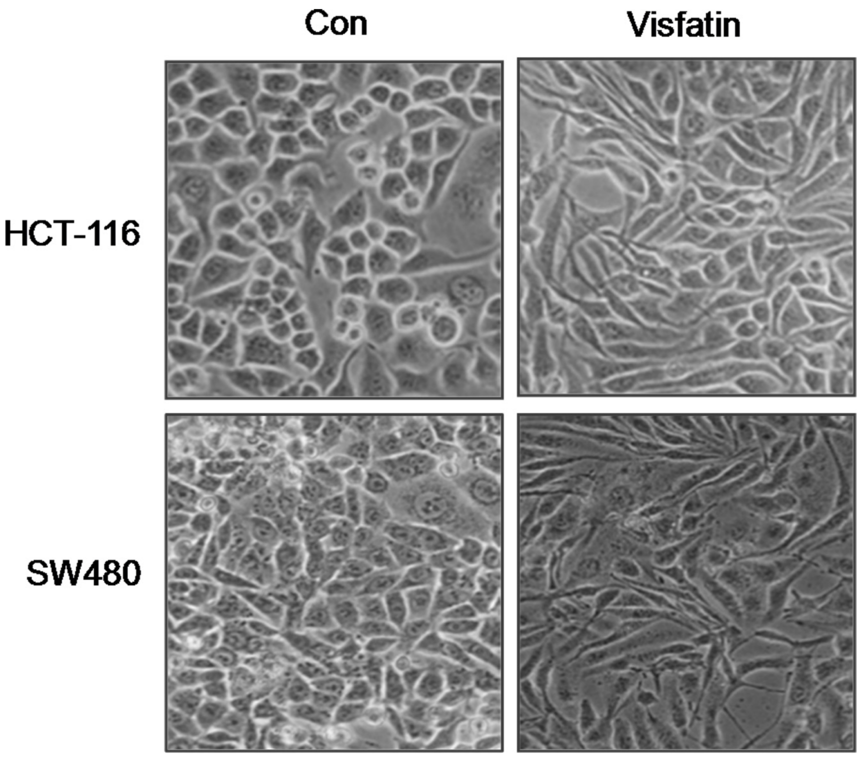

Supplementary Figure S1: CRC cells were treated with 200ng/ml visfatin for 48 h. Cell morphological changes associated with EMT are shown in the phase contrast image.

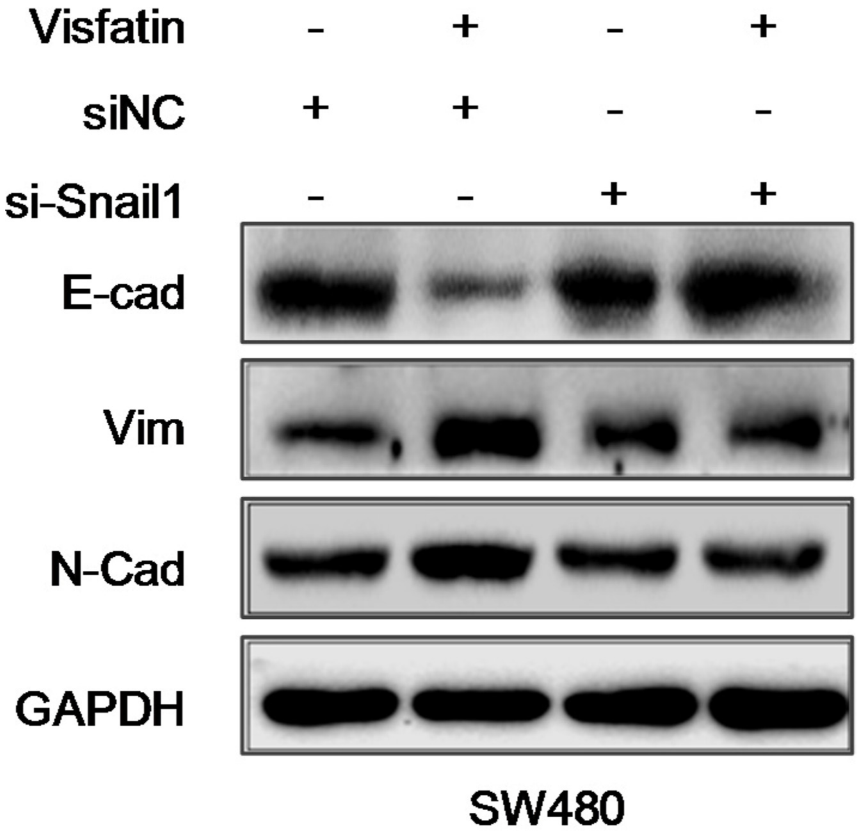

Supplementary Figure S2: SW480 cells transfected with Snail specific si-RNA (si-Snail1) or negative control si-RNA (si-NC) for 24 h and then exposed to 200 ng/ml visfatin for another 48 h, the expression of EMT related markers were measured by use of Western blot analysis.

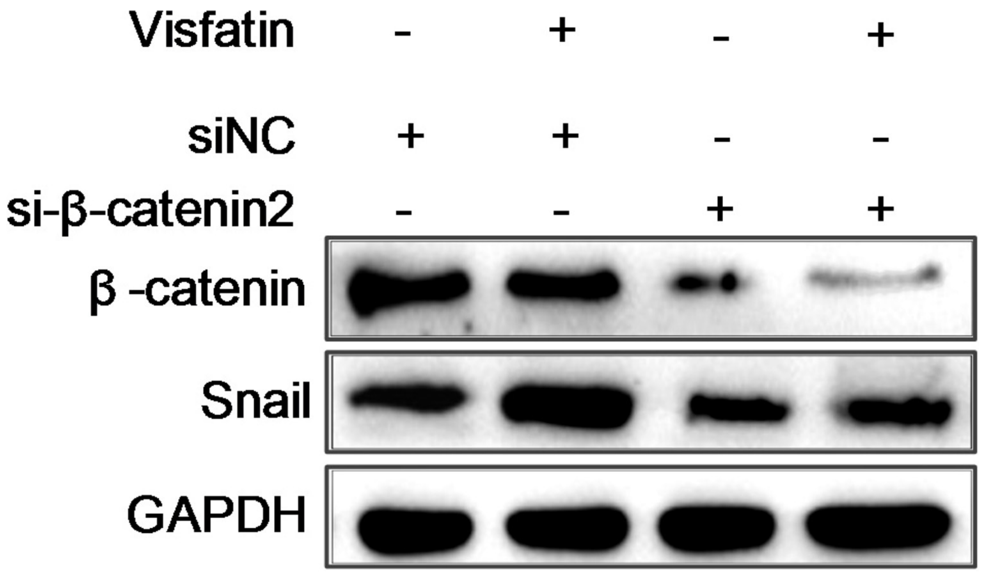

Supplementary Figure S3: HCT-116 cells were transfected with si-β-catenin2 or si-NC for 24 h, and then treated with 200ng/ml visfatin for 12 h.

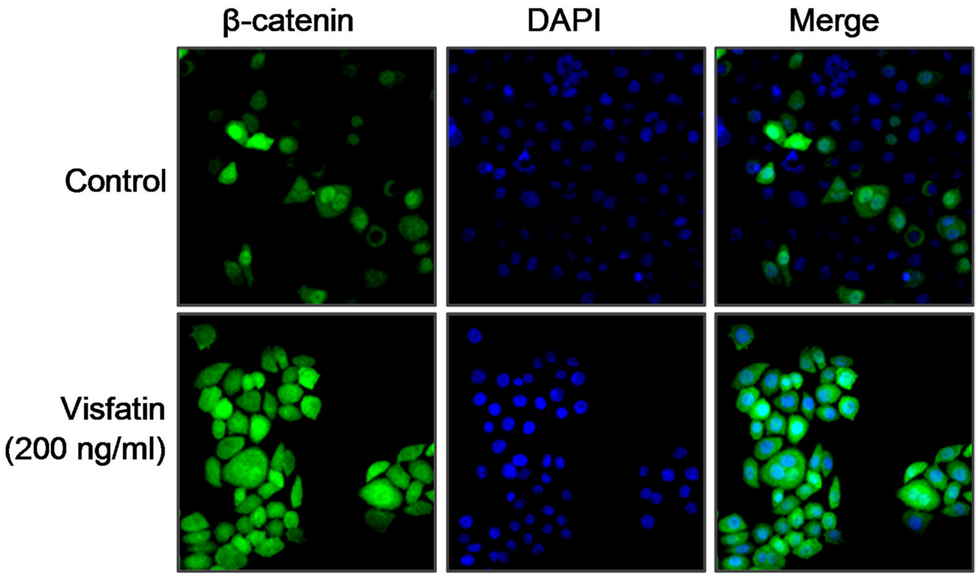

Supplementary Figure S4: HCT-116 cells were treated with 200 ng/ml visfatin for 24 h, the cellular location of β-catenin (green) were examined by immunofluorescence staining and nuclei were stained with DAPI (blue).
